# Natural Images Allow Universal Adversarial Attacks on Medical Image Classification Using Deep Neural Networks with Transfer Learning

**DOI:** 10.3390/jimaging8020038

**Published:** 2022-02-04

**Authors:** Akinori Minagi, Hokuto Hirano, Kauzhiro Takemoto

**Affiliations:** Department of Bioscience and Bioinformatics, Kyushu Institute of Technology, Iizuka 820-8502, Fukuoka, Japan; minagi.akinori731@mail.kyutech.jp (A.M.); hirano.hokuto346@mail.kyutech.jp (H.H.)

**Keywords:** deep neural networks, transfer learning, medical imaging, adversarial attacks, security, privacy

## Abstract

Transfer learning from natural images is used in deep neural networks (DNNs) for medical image classification to achieve a computer-aided clinical diagnosis. Although the adversarial vulnerability of DNNs hinders practical applications owing to the high stakes of diagnosis, adversarial attacks are expected to be limited because training datasets (medical images), which are often required for adversarial attacks, are generally unavailable in terms of security and privacy preservation. Nevertheless, in this study, we demonstrated that adversarial attacks are also possible using natural images for medical DNN models with transfer learning, even if such medical images are unavailable; in particular, we showed that universal adversarial perturbations (UAPs) can also be generated from natural images. UAPs from natural images are useful for both non-targeted and targeted attacks. The performance of UAPs from natural images was significantly higher than that of random controls. The use of transfer learning causes a security hole, which decreases the reliability and safety of computer-based disease diagnosis. Model training from random initialization reduced the performance of UAPs from natural images; however, it did not completely avoid vulnerability to UAPs. The vulnerability of UAPs to natural images is expected to become a significant security threat.

## 1. Introduction

Transfer learning from natural image datasets (e.g., the ImageNet dataset [1]) is a widely used technique in deep neural networks (DNNs) for image classification and has been applied well to medical imaging in particular [2]. Although the amount of medical image data is often limited, transfer learning enables the acquisition of highly accurate DNNs from such limited image data by fine-tuning existing model architectures (e.g., Inception V3 [3] and ResNet50 [4]) pretrained on the ImageNet dataset. Transfer learning techniques have been used for medical image classification (e.g., skin cancer classification using photographic images [5], retinal disease classification using optical coherence tomography (OCT) images [6], and pneumonia classification based on chest X-ray images [6]). The high diagnostic performance of these DNNs is equivalent to that of healthcare professionals [7]. Thus, DNNs with transfer learning are being used for medical image diagnosis to achieve faster and more reliable decision-making in clinical environments [2].

However, the practical application of DNNs to disease diagnosis may still be debatable owing to the existence of adversarial examples [8,9,10], which are input images contaminated with small specific perturbations that cause misclassifications by DNNs. Given that diagnosing disease involves making high-stake decisions, the existence of adversarial examples is a security concern [11]. Adversarial examples likely cause a misdiagnosis and various social disturbances [12] and limit deep learning applications under both safety- and security-critical environments [13]. Therefore, it is also important to evaluate the reliability and safety of DNNs against adversarial attacks in medical imaging.

Many previous studies demonstrated that DNN models are vulnerable to input-dependent adversarial attacks, in which an individual adversarial perturbation is used to generate adversarial examples against each input image in skin cancer [12] and pneumonia classifications [14]. More importantly, a previous study [15] showed that a small single perturbation called universal adversarial perturbation (UAP) [16,17] can cause the failure of most DNN-based image classification tasks to become a security threat when applying a DNN-based medical image classification. UAP-based attacks are more realistic because they are image agnostic. Adversaries can more easily implement adversarial attacks in real-world environments with lower computational costs [16].

A simple way to avoid an adversarial attack is to render training data and any other similar publicly unavailable domain-specific data (e.g., medical images in the case of medical image classification) because various methods of adversarial attacks [8,9,10] (from attack methods that assume access to DNN model weights to those that do not) generally assume the use of such data to generate adversarial perturbations. Given that the data availability of medical images is generally limited in terms of security and privacy preservation [11], adversarial attacks on DNN-based medical image classifications are limited. However, we doubt this prediction owing to the properties of transfer learning for medical imaging [18]. Specifically, transfer learning considers that model weights pretrained with the ImageNet dataset (natural images) are fine-tuned with medical images; however, fine-tuned DNN models for medical imaging are known to be similar to the original pretrained DNN models, despite the fine-tuning process. In addition, larger DNN models do not change through training. It seems that DNN models obtained by fine-tuning well-used model architectures (e.g., Inception V3 and ResNet50) with medical images show similar reactions to both medical and natural images.

Thus, we developed and tested the hypothesis that adversarial perturbations against fine-tuned DNN models (Section 2) are generatable using not only training data (medical images) but also natural images (e.g., the ImageNet dataset) (Section 3.1 and Section 3.2). Following our previous study [15], we considered representative medical image classifications (skin cancer classification [5], retinal disease classification [6], and pneumonia classification [6]) and investigated the vulnerability of fine-tuned DNN models with several architectures to adversarial perturbations generated using natural images. In this study, we focus on universal adversarial attacks [16,17] rather than input-dependent adversarial attacks. This is because the input-dependent adversarial attacks are less effective; in particular, it is costly to determine the medical images that result in a misclassification from an adversarial perturbation generated using a natural image. By contrast, UAPs (generated using natural images) can be used for any medical image because they are image agnostic. To evaluate the effects of transfer learning on vulnerability to UAPs, we also considered the DNN model architecture training from random initialization (Section 3.3).

## 2. Materials and Methods

### 2.1. Medical Image Datasets and Models

We used the medical image datasets and DNN models previously described in [15] (see also github.com/hkthirano/MedicalAI-UAP). A brief description is provided below.

For skin cancer classification, we used skin lesion images consisting of 7000 training images and 3015 test images that were classified into seven classes: melanoma (MEL), melanocytic nevus (NV), basal cell carcinoma, actinic keratosis/Bowens disease (intraepithelial carcinoma), benign keratosis (solar lentigo/seborrheic keratosis/lichen planus-like keratosis; BKL), dermatofibroma, and vascular lesions. For retinal disease classification, we used OCT images consisting of 7840 training and 3360 test images classified into four different classes, i.e., choroidal neovascularization with a neovascular membrane and associated subretinal fluid (CNV), diabetic macular edema with retinal-thickening-associated intraretinal fluid, multiple drusen found in early age-related macular degeneration (DRUSEN), and in a normal retina with preserved foveal contour and lack of retinal fluid/edema (NM). For pneumonia classification, we used chest X-ray images consisting of 1800 training and 540 test images classified into binary classes of no pneumonia (NORMAL) or viral or bacterial pneumonia (PNEUMONIA). Please note that the OCT and chest X-ray image datasets were class-balanced, whereas the skin lesion image dataset was not (see [15] for details).

Following previous studies, the Inception V3 architecture [3] was mainly considered [5,6]. To evaluate how the model architecture affects the vulnerability to UAPs, we also used the VGG16 [19] and ResNet50 [4] architectures. These DNN model architectures pretrained using the ImageNet dataset were fine-tuned with the training images in a medical image dataset, using the learning rate schedule and data augmentation (see [15] for the test accuracies of these models). To evaluate the effects of transfer learning on the vulnerability to UAPs from natural images, we also obtained the Inception V3 models trained by applying the training images in each medical image dataset from random initialization, and the training conditions in this case (e.g., the learning rate schedule and condition of data augmentation) were identical to those in the case of transfer learning, except for the number of epochs and random initialization. Given that transfer learning contributes to a faster convergence [18], more epochs may be required when training the models from random initialization. Thus, we here set the number of epochs to 300 (six times as large as that for transfer learning).

### 2.2. Universal Adversarial Perturbations and Natural Images

Following our previous study [15], we used simple iterative algorithms [16,17] to generate the UAPs. We considered both non-targeted attacks, which cause a misclassification (i.e., a task failure resulting in an input image being assigned an incorrect class), and targeted attacks, which cause a DNN to classify an input image into a specific class. For the non-targeted UAPs, the Adversarial Robustness 360 Toolbox (ART) [20] (version 1.0; github.com/Trusted-AI/adversarial-robustness-toolbox, accessed on 18 November 2021) was used. For the targeted UAPs, we used our proposed method [17] (see also github.com/hkthirano/targeted_UAP_CIFAR10, accessed on 18 November 2021), which is a modified version of the non-targeted UAP algorithm [16].

The algorithms apply a classifier and generate UPAs ρ from a set of input images X, under the constraint in which the $L_{p}$ norm of the perturbation $\|\rho {\|}_{p}\leq \xi$ for a small ξ value. The algorithms begin with ρ=0 (no perturbation) and iteratively update ρ by additively obtaining an adversarial perturbation for an input image x, selected randomly from X without replacement through the fast gradient sign method [8] with the attack strength parameter ϵ. These iterative updates continue until the number of iterations reach the maximum $i_{\max}$.

Using these algorithms, UAPs against medical DNN models were generated using natural images. The algorithms originally assume that X corresponds to the training dataset (e.g., medical images) to generate the UAPs; however, in this study, we used natural images instead of medical images. Specifically, we used the training images in the ImageNet dataset because the DNN models were pretrained using the ImageNet dataset. The ImageNet training set was downloaded from www.image-net.org/download.php (accessed on 17 June 2020). Moreover, we also considered the Open Images dataset (V6), a different dataset of natural images, to evaluate the dataset dependency in the performance of the UAPs. The dataset was downloaded from storage.googleapis.com/openimages/web/download.html (accessed on 22 November 2020). For each dataset, 100,000 randomly selected images were used to generate the UAPs. The images were gray-transformed when generating UAPs against the DNN models for referable diabetic retinopathy and pneumonia classifications.

For both skin lesion and chest X-ray image classifications, the parameters ϵ and p were set to 0.0005 and 2, respectively. For the OCT image classification, ϵ and p were set to 0.0013 and ∞, respectively. However, a different ϵ was considered for the Inception V3 models trained from random initialization. When generating UAPs using training images, ϵ was 0.0044, 0.0036, and 0.0066 for the skin lesion, OCT, and chest X-ray image classifications, respectively. When generating UAPs using natural images, ϵ was 0.0050, 0.0020, and 0.0026 for the skin lesion, OCT, and chest X-ray image classifications, respectively. The parameters ϵ and p were selected using a grid search to maximize the performance of the UAPs (see below) for the input images. The parameter $i_{\max}$ was set to 1. The parameter ξ was set based on the ratio ζ of the $L_{p}$ norm of the UAP to the average $L_{p}$ norm of an image in the dataset (see [15] for the actual values of the average $L_{p}$ norms).

To compare the performance of the UAPs between the training and natural images, we also obtained the UAPs generated using the training datasets (medical images) from our previous study [15]. Random vectors (random UAPs) sampled uniformly from a sphere of a specified radius were used to compare the performance of the generated UAPs with those of the random controls [16].

### 2.3. Evaluating the Performance of UAPs

The performance evaluation the of UAPs was based on the procedures established in our previous study [15]. Both the fooling rate $R_{f}$ and targeted attack success rate $R_{s}$ were used to evaluate the performance of a non-targeted UAP ($\rho_{\mathrm{nt}}$) and targeted UAP ($\rho_{t}$). $R_{f}={\left|X\right|}^{-1}\sum_{x\in X}I\left(C\left(x\right)\neq C\left(x+\rho_{\mathrm{nt}}\right)\right)$, where C(x) is the output (class or label) of a classifier (DNN) for an input image x in an image set X. Function I(A) takes a value of 1 if condition A is true, and 0 otherwise. Here, $R_{f}$ indicates the fraction of adversarial images from which the labels predicted are inconsistent with the labels predicted from clean images to all images in the set. In addition, $R_{s}={\left|X\right|}^{-1}\sum_{x\in X}I\left(C\left(x+\rho_{t}\right)=y\right)$, indicating the proportion of adversarial images classified into target class y to all images in set X. As mentioned in our previous study [15], $R_{s}$ has a baseline $R_{s}$ observed without UAPs. The $R_{s}$ baselines of UAPs targeted to a specified class were ~25% and ~50%, respectively, for the OCT and chest X-ray image datasets. For the skin lesion dataset, the $R_{s}$ baselines of UAPs targeted to MEL and NV were ~10% and ~65%, respectively. In addition, $R_{f}$ and $R_{s}$ were computed using test images from the medical image dataset. The confusion matrixes on test images from the medical image dataset were also obtained for evaluating the transition in prediction owing to the UAPs for each class. The row-normalized confusion matrixes were obtained to account for imbalanced datasets.

## 3. Results

### 3.1. Natural Images Allow Non-Targeted Universal Adversarial Attacks on Medical Image Classification

**Figure 1:**
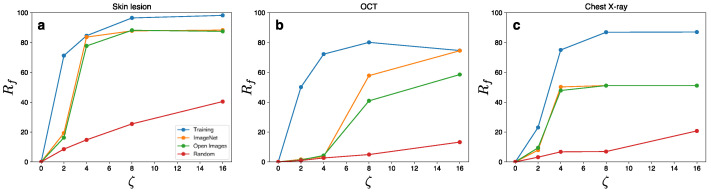
Vulnerability to non-targeted UAPs generated using training (medical image), ImageNet, and Open Images datasets. Line plots of the fooling rate $R_{f}$ (%) against Inception V3 model versus perturbation magnitude ζ (%) for the skin lesion (**a**), OCT (**b**), and chest X-ray (**c**) image classifications. The legend label denotes the set of input images used to generate the UAPs, except for “Random”, which indicates random UAPs.

**Figure 2:**
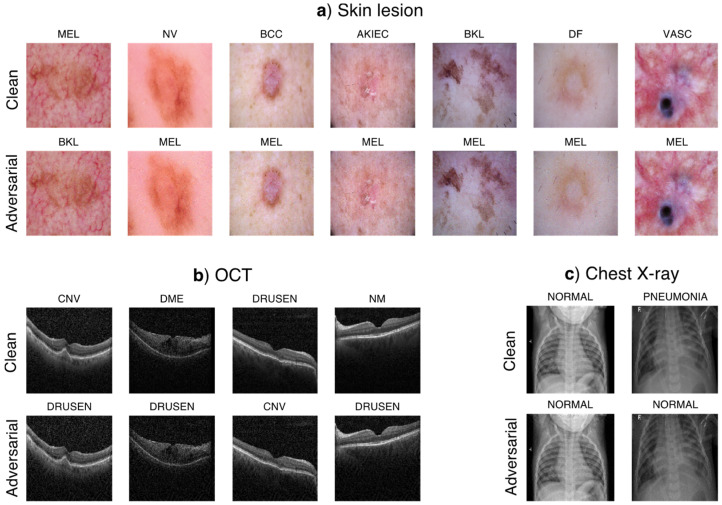
Clean images and their adversarial examples generated using non-targeted UAPs from the ImageNet dataset, against the Inception V3 model for the skin lesion (**a**), OCT (**b**), and chest X-ray (**c**) image classifications. ζ=4% in (**a**,**c**) and ζ=8% in (**b**). Labels next to the images are the predicted classes. The clean (original) images are correctly classified into their actual labels.

We first consider the Inception V3 models as they were used in previous studies on DNN-based medical imaging [5,6] and evaluated whether non-targeted UAPs against the medical DNN models are generatable using natural images (Figure 1). The performance of the UAPs generated using the natural images was less effective than that of the UAPs generated in the training datasets (medical images); specifically, the UAPs from the training images achieved a higher fooling rate $R_{f},$ with a smaller perturbation magnitude ζ, in comparison to the UAPs from the natural images. However, $R_{f}$ of the UAPs generated using the natural images was significantly higher than that of random UAPs; moreover, they also increased rapidly with ζ and reached a high $R_{f}$, despite a low ζ. Specifically, $R_{f}$ ~80% and ~50% were achieved at ζ=4% for the skin lesion (Figure 1a) and chest X-ray image classifications (Figure 1c), respectively. In addition, $R_{f}$ was 40–60% at ζ=8% for the OCT image classification (Figure 1b). These UAPs were almost imperceptible. As a representative example, clean images and their adversarial examples owing to the UAPs from the ImageNet dataset are shown in Figure 2. The adversarial examples owing to the UAPs from the training and open image datasets are shown in Figures S1–S3 in File S1. These results indicate that small UAPs from natural images also cause a misclassification of DNN-based medical image classifications. We also found that the performance of UAPs from natural images has no strong dataset dependency because $R_{f}$ values of the UAPs from the Open Images dataset were almost similar to those of the UAPs generated using the ImageNet dataset, although small differences in $R_{f}$ were observed, i.e., ~40% and ~60% for the Open Images and ImageNet datasets, respectively.

**Figure 3:**
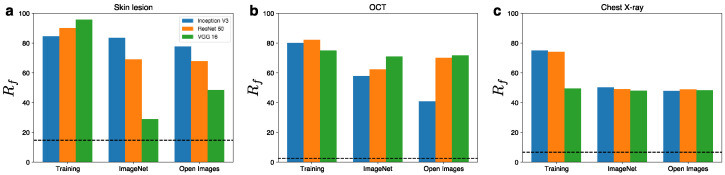
Difference in the fooling rate $R_{f}$ (%) of the UAPs according to the model architectures for skin lesions (**a**), OCT (**b**), and chest X-ray (**c**) image classifications. ζ=4% in (**a**,**c**) and ζ=8% in (**b**). Dashed lines indicate $R_{f}$ (%) of random UAPs (random controls).

For the ResNet50 and VGG16 models, $R_{f}$ of the UAPs from the natural images was also significantly higher than that of the random control (Figure 3), although it was less than that of the UAPs from the training images. However, $R_{f}$ at the same ζ was different between the model architectures, except for the chest X-ray image classification. For the skin lesion image classification (Figure 3a), $R_{f}$ of the UAPs with ζ=4% was approximately 80% for the Inception V3 model, whereas it was lower for the ResNet50 and VGG16 models. Specifically, $R_{f}$ against the ResNet50 and VGG models was approximately 70% and 30–50%, respectively. For the OCT image classification (Figure 3b), a slightly higher $R_{f}$ (60–70%) of the UAPs against the ResNet50 and VGG16 models with ζ=8% was observed, in comparison to the Inception V3 model (40–60%). For the chest X-ray image classification (Figure 3c), $R_{f}$ of the UAPs with ζ=4% from the natural images was ~50%, independent of the model architecture.

**Figure 4:**
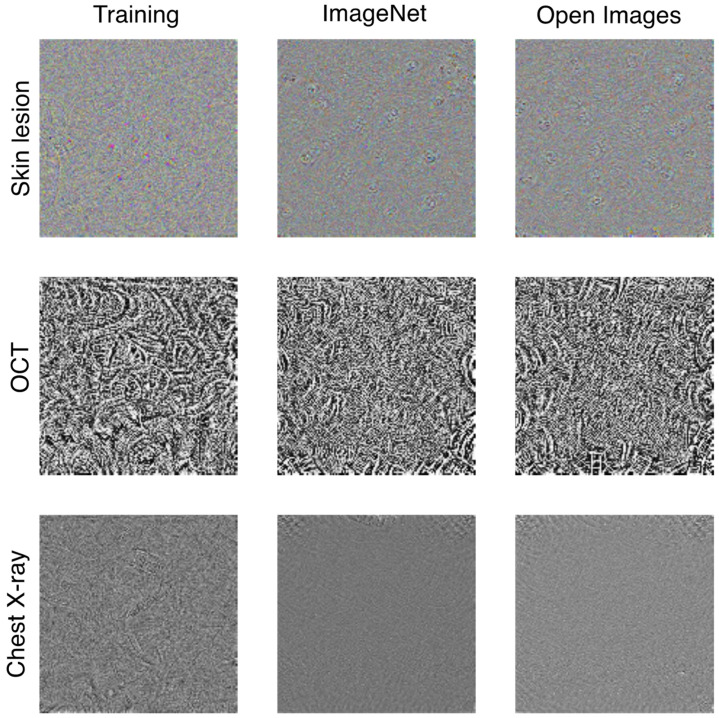
Visualization of non-targeted UAPs generated using training (medical image), ImageNet, and Open Images datasets against Inception V3 models for skin lesion, OCT, and chest X-ray image classifications. UAPs are visually emphasized for clarity; specifically, each UAP is scaled by a maximum of 1 and minimum of zero.

**Figure 5:**
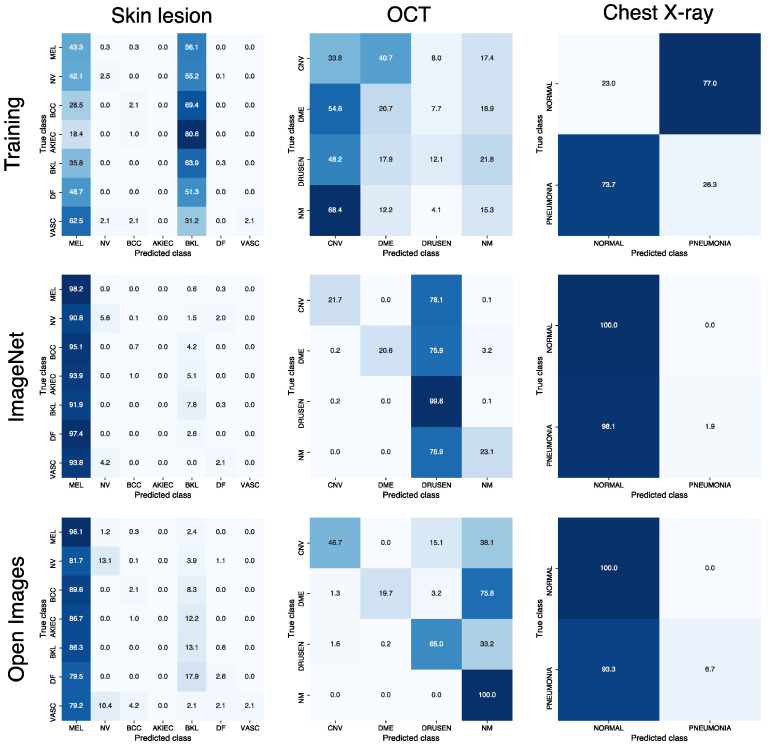
Normalized confusion matrixes applied to Inception V3 models attacked using non-targeted UAPs from training, ImageNet, and Open Images datasets for skin lesion, OCT, and chest X-ray image classifications.

As expected from the observed difference in $R_{f}$ between the UAPs from the training images and those generated from natural images, those from the natural images were visually different from those from the training images for the same ζ. Figure 4 shows the UAPs generated using the training, ImageNet, and Open Images datasets against the Inception V3 models. Moreover, Figure 5 also shows a different tendency of misclassification of the DNN models (Inception V3 models) owing to the different UAPs between those from the natural images and those from the training images, although the confusion matrix patterns are similar in that dominant classes are observed (i.e., most images are classified into a small number of specific classes owing to the UAPs). For the skin lesion image classification, the dominant classes were MEL and BKL when using the UAPs from the training images; however, the dominant class was only MEL when using the UAPs from the natural images (both the ImageNet and Open Images datasets). For the OCT image classification, the dominant class was CNV in the case of the UAPs from the training images; however, it was DRUSEN and NM in the case of the UAPs from the ImageNet dataset and in the case of the UAPs from the Open Images dataset. For classification of chest X-ray images, the DNN model almost perfectly misclassified the test images because of the UAPs from the training images; however, it classified most of the images into NORMAL because of the UAPs from the natural images (both the ImageNet and Open Images datasets), indicating that $R_{f}$ saturated at ~50% (Figure 1c).

The dominant classes might differ based on the model architecture and natural image datasets, except for the chest X-ray image classification. For the skin lesion classification, the dominant class was BKL for the UAPs from both the ImageNet and Open Images datasets against the VGG16 model and for the UAP from the Open Images dataset against ResNet50, whereas it was MEL for the UAPs from the ImageNet dataset against the ResNet50 model (Figure S4 in File S1). For the OCT image classification, the dominant classes of the UAPs from both the ImageNet and Open Images datasets were DRUSEN for ResNet50; however, they were CNVs for the VGG16 model (Figure S5 in File S1). For the chest X-ray image classification, the dominant classes were NORMAL, independent of the model architectures and natural image datasets (Figure S6 in File S1).

### 3.2. Natural Images Allow Targeted Universal Adversarial Attacks on Medical Image Classification

We also investigated the vulnerability of the medical DNN models to the targeted UAPs generated from natural images. Following our previous study [15], targeted attacks were considered the most significant case and were the control in each medical image dataset. The most significant cases correspond to MEL, CNV, and PNEUMONIA in the skin lesion, OCT, and chest X-ray image datasets, respectively. The controls correspond to NV, NM, and NORMAL in the skin lesion, OCT, and chest X-ray image datasets, respectively. Table 1 shows the success rate $R_{s}$ of a target attack of the UAPs against the DNN models. The UAPs were extremely small and almost imperceptible because ζ=4% in the skin lesion and chest X-ray image classifications and ζ=8% in the OCT image classification, as in the case of the non-targeted UAPs (see Figure 2). However, overall, the values of $R_{s}$ (>90%) of the UAPs from both the ImageNet and Open Images datasets were significantly higher than those of the random UAPs, and were mostly similar to those of the UAPs from the training datasets (medical images). This tendency is independent of the model architecture. However, a low $R_{s}$ was observed in a small number of cases. The values of $R_{s}$ of the UAPs from the ImageNet and Open Images targeted to MEL were ~10%, which were mostly similar to the random control for the ResNet50 model, whereas they were ~95% for the Inception V3 and ResNet50 models. The values of $R_{s}$ of the UAPs from the ImageNet and Open Images targeted to CNV were 35–50%, which were higher than random controls for the ResNet50 model, whereas they were ~100% for Inception V3 and VGG16 models. Finally, the values of $R_{s}$ of the UAPs from the ImageNet and Open Images targeted to PNEUMONIA were 60–80%, which was higher than that of the random controls, whereas that of the UAPs from the training images was ~100%.

**Figure 6:**
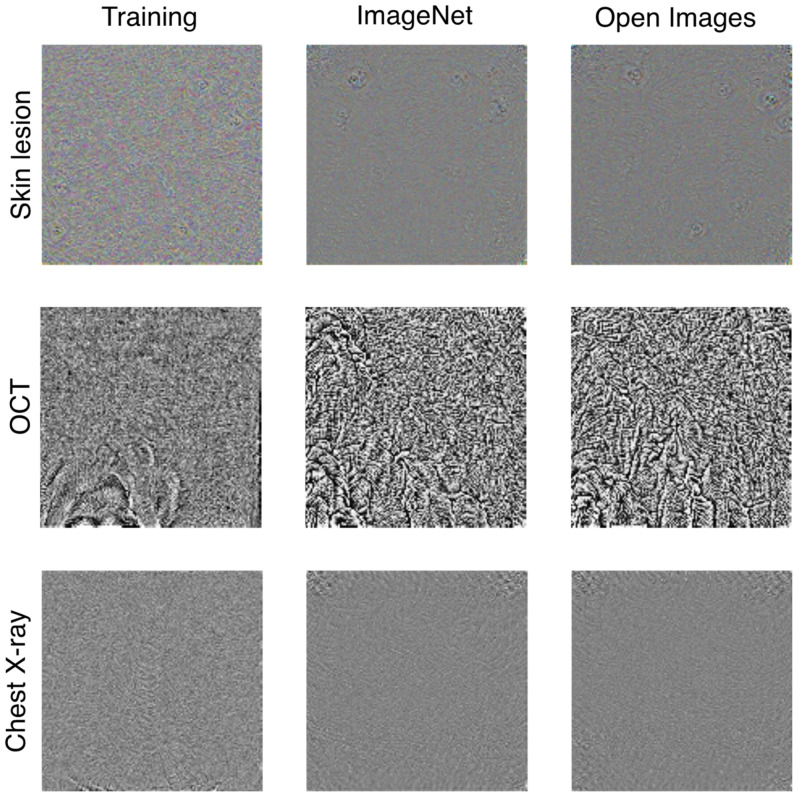
Visualization of targeted UAPs generated using training (medical image), ImageNet, and Open Images datasets against Inception V3 models for skin lesion, OCT, and chest X-ray image classifications. UAPs are visually emphasized for clarity; in particular, each UAP is scaled by a maximum of 1 and minimum of zero.

As representative examples, Figure 6 shows the UAPs generated using several image datasets for targeted attacks on MEL, CNV, and NORMAL against the Inception V3 models. These UAPs showed an $R_{s}$ value of ~100%; however, the UAPs from natural images were visually different from those from the training images for each medical image dataset.

### 3.3. Effect of Transfer Learning on Vulnerability of the UAPs from Natural Images

It is predicted that transfer learning from natural images (the ImageNet dataset, in particular) causes the observed vulnerability of the UAPs from natural images to DNN-based medical image classification. To test this more deeply, we considered the Inception V3 models, which are widely used in medical image classification [5,6], which were trained with the training images in each medical image dataset from a random initialization. For the datasets of skin lesion, OCT, and chest X-ray images, the test accuracies of the models were 79.2%, 95.3%, and 97.8%, respectively. The accuracies of the models trained from a random initialization were mostly similar to those (95.5% and 97.6%, respectively [15]) of the models trained from transfer learning for the OCT and chest X-ray image datasets; however, the accuracy from random initialization was slightly lower than that (87.7% [15]) from transfer learning for the datasets on skin lesion images.

We evaluated the vulnerability of non-targeted UAPs against these Inception V3 models (Table 2) and found that the UAPs from natural images were less effective for fooling the DNN-based medical image classifications. For the skin lesion image classification, the $R_{f}$ value of the UAP from the ImageNet dataset was only ~50%, despite a larger ζ (ζ=8%, i.e., twice larger than the case shown in Figure 3a), whereas $R_{f}$ of the UAP from the training images was ~90%. For the chest X-ray image classification, $R_{f}$ of the UAP from the ImageNet dataset was only ~20% despite a larger ζ (ζ=8%, i.e., twice larger than the case shown in Figure 3c), whereas $R_{f}$ of the UAP from the training images was ~45%. The results indicate that model training from random initialization reduces the performance of the UAPs from natural images. However, the vulnerability of the UAPs from natural images is not completely avoided because of random initialization. The value of $R_{f}$ of the UAPs from the ImageNet dataset was still larger than that of the random UPAs, and was mostly similar between the UAPs from the ImageNet dataset and the UAPs from the training images for the OCT image classification, although ζ=16% (i.e., twice larger than the case shown in Figure 3b).

## 4. Discussion

We hypothesized that the UAPs against DNN models with transfer learning are generatable using not only training datasets (medical images) but also natural images because pretrained models do not change significantly after fine-tuning. We further demonstrate that fine-tuned models for medical image classification are vulnerable to both non-targeted and targeted UAPs from natural images (Figure 1 and Table 1). Vulnerability was confirmed in several of the model architectures and thus might be a universal aspect of a DNN. Given the fact that the medical DNN models with transfer learning from the ImageNet dataset are vulnerable to not only UAPs from the ImageNet dataset but also to UAPs from the Open Images datasets, this vulnerability to the UAPs may be independent of the natural image datasets, indicating that the UAPs against the DNN models with transfer learning are generatable using any publicly available natural images. This may be a novel security threat to a DNN-based medical image diagnosis, in particular, it indicates that mostly imperceptible UAPs are generatable without trained medical data or any other similar medical data (regardless of how much such data are kept a secret). Unlike the prediction that adversarial attacks on DNN-based medical image classifications are difficult because the data availability of medical images is generally limited in terms of security and privacy preservation, the results show that medical DNN models are easier to fool. Adversaries can disrupt medical image diagnoses based on DNN models, even if they never access such medical data.

The UAPs from natural images seem to differ with those from the training (medical) images (Figure 4 and Figure 6), and the characteristics (e.g., $R_{f}$, dominant classes, and $R_{s}$) of the UAPs from natural images were partly different from those of the UAPs from the training images. This may be because of the difference in the composition of the predicted labels between the training and natural images (Tables S1–S3 in File S1). For chest X-ray image classification, for example, ~80% of both the ImageNet and Open Images datasets were classified as PNEUMONIA regardless of the model architecture (Table S3 in File S1), whereas the training images were mostly class-balanced. Because the non-targeted attack algorithm [16] considers maximizing $R_{f}$, a large $R_{f}$ is achieved when images with such an abundant label are misclassified. In contrast, misclassifying images with less-abundant labels has little advantage for maximizing $R_{f}$. The performance of non-targeted UAPs is less effective (images with less-abundant labels are difficult to fool), and less-abundant labels tend to correspond to dominant classes when the predicted labels of natural images are imbalanced. For the chest X-ray image classification, the dominant class of the UAPs from natural images was NORMAL (Figure 5); as a result, $R_{f}$ was saturated at ~50% (Figure 1c). The tendency of the dominant classes to correspond to the less-abundant predicted labels (see Tables S1 and S2) was also observed for the skin lesion and OCT image classification (Figure 5). The imbalanced predicted labels of the natural images also affect the performance of the targeted UAPs. Because the targeted attack algorithm [17] considers maximizing $R_{s}$, a large $R_{s}$ will have already been achieved for targeted attacks to an abundant label in a dataset. Thus, UAPs are rarely updated in the iterative algorithm; as a result, $R_{s}$ rarely increases. The targeted attacks on NM and PNEUMONIA, which are the abundant labels in the dataset (Tables S2 and S3 in File S1), were less effective respectively for the OCT and chest X-ray image classifications (Table 2). The performance of the UAPs from natural images may increase by controlling the composition of the predicted labels of the natural images (e.g., using data augmentation).

However, more careful examinations are required to reveal what happens inside the DNN models due to UAPs and how the effects of UAPs from natural images on the DNN models are different compared to UAPs from medical images. In this context, it might be useful for investigating how explainability [21] in DNN models alters due to UAPs, given that their techniques are typically used in medical imaging applications [22]. Explainability methods, e.g., Gradient class activation mapping (Grad-CAM) [23], provide saliency maps that indicate the importance of each pixel in the input images for the model outputs. The differences in the saliency maps might be helpful for evaluating the effects of UAPs on DNN models.

This study showed that the UAPs were generatable without training data. In this context, UAPs from natural images are regarded as black-box attacks. However, UAPs are not complete black-box attacks because they assume a white-box condition, i.e., the model parameters (e.g., the gradient of the loss function) are accessible. This is because the well-used UAP algorithms [16,17], which we also used, are limited to the white-box condition. However, this limitation poses a few problems for adversaries. As represented by COVID-Net [24], a DNN model for COVID-19 detection from chest X-ray images, DNN models are often developed as open-source projects by expecting that many people, including researchers and citizens data scientists, will accelerate the development of high-performance DNN-based systems. Moreover, collaboration among multiple institutions is required to develop DNN models with a high diagnostic performance and the distribution of deep learning models has been proposed as an effective alternative to the sharing of patient data [25]. Even if model parameters (e.g., weights and the loss gradient) are not accessible, they may be estimated [26] because DNN-based medical imaging is frequently developed through a fine-tuning of the existing pretrained models, such as Inception, ResNet, and VGG, as considered in this study. Because DNNs are aimed at real-world usage (e.g., automated support for clinical diagnosis), the assumption that adversaries cannot access DNN models may be unrealistic.

Nevertheless, our findings may also be useful for developing black-box attack methods that generate adversarial perturbations based on only the model outputs (e.g., confidence scores). Several methods for black-box attacks have been proposed [27,28,29,30]. Although they are limited to input-dependent adversarial attacks, universal adversarial attacks may be possible under the black-box condition because CNNs are sensitive to the directions of the Fourier basis functions [31]. However, these methods assume the use of domain-specific data (e.g., medical images in the case of medical image classification) that are not included in the training data. Our study indicates that this assumption was not required. Adversaries may be able to apply black-box attacks more easily than previously thought, simply using natural images instead of domain-specific images.

A simple solution for avoiding the vulnerability of UAPs from natural images is to train DNN models from random initialization (i.e., without pretrained weights). The performance of UAPs from natural images was overall lower in the DNN model trained with random initialization (Table 2), compared to the DNN models with transfer learning. This might be because the model weights differ from the pretrained weights from the natural images. However, training from random initialization does not completely prevent the vulnerability of UAPs from natural images. As shown in Table 2, the performance of the UAPs was still higher than that of random controls; moreover, it was almost similar to that of the UAPs from training images in certain cases (e.g., OCT image classification). In addition, trade-offs with the prediction performance must be considered. Because transfer learning contributes to a faster convergence [18], the prediction performance may decrease when training DNN models from random initialization, in comparison to transfer learning, when considering the same number of training steps (epochs); thus, this solution may be unrealistic in terms of the practical desire to achieve a high prediction performance with a lower computational cost.

Given the vulnerability resulting from the discrepancy in learned features between the natural (e.g., the ImageNet dataset) and medical images, another solution to avoid a vulnerability may be to use a transfer learning approach in which a DNN model pretrained using a large number of unlabeled medical images is used to train the DNN model on a relatively small number of labeled medical images [32] (i.e., transfer learning using self-supervised models [33]). Although this study did not evaluate this type of transfer learning because the amount of medical image data is limited, it would be interesting to investigate the extent to which vulnerability is avoided through the use of self-supervised models.

Adversarial defenses [34] also need to be considered to reduce vulnerability to UAPs. Although recent developments in adversarial defenses [35,36,37,38] have been remarkable, comprehensive comparative evaluations [39,40] showed that promising defense methods are less effective than reported. Explainability (e.g., saliency maps from Grad-CAM) might be a useful indicator for determining adversarial attacks. The saliency maps of adversarial images are expected to differ from those of clean images [41,42]. However, explainability-based defenses might be limited since Grad-CAM could be easily deceived [43]; specifically, adversaries could adjust DNN models to allow Grad-CAM to yield their desired saliency maps. Defending against adversarial attacks becomes a type of cat-and-mouse game [12], indicating the need for continued development of adversarial defense methods. Further investigation is needed to evaluate how much novel methods (e.g., adversarial querying [44], for producing adversarially robust meta-learners) reduce vulnerability to UAPs.

## 5. Conclusions

Our study showed that natural images allow universal adversarial attacks on medical image classification using deep neural networks with transfer learning. It was expected that adversarial attacks are limited because medical images used for training are generally unavailable; however, existing algorithms can generate UAPs using natural images instead of training datasets (medical images). Transfer learning from natural images is widely used for medical imaging because the amount of medical image data is often limited. However, the use of transfer learning causes a security hole, therefore reducing the reliability and safety of computer-based disease diagnosis. Our findings demonstrate a novel vulnerability of DNNs to adversarial attacks and can assist in an increase in the security of such networks. They are particularly useful for designing operation strategies for medical DNNs.

## Figures and Tables

**Table 1:** Targeted attack success rates $R_{s}$ (%) of targeted UAPs against Inception V3, ResNet50, and VGG16 models for each target class. ζ=4% for the skin lesions and chest X-ray image classifications, and ζ=8% for the OCT image classification. The column “UAP” indicates which input image set was used to generate the UAP, except for “Random”, which indicates the use of random UAPs.

Medical Images	Target Class	UAP	Model Architecture		
			Inception V3	ResNet50	VGG16
Skin lesion	NV	Training	97.9	99.2	98.7
		ImageNet	98.8	96.2	86.6
		Open Images	99.1	94.5	86.9
		Random	64.1	70.2	73.3
	MEL	Training	97.1	97.7	97.6
		ImageNet	97.1	96.0	10.5
		Open Images	96.6	94.5	10.4
		Random	14.5	11.8	8.8
OCT	NM	Training	98.2	99.4	98.6
		ImageNet	98.2	99.7	92.3
		Open Images	99.4	99.8	94.0
		Random	27.6	29.3	26.5
	CNV	Training	99.3	99.7	99.9
		ImageNet	99.2	35.5	98.3
		Open Images	99.3	48.3	96.2
		Random	26.5	26.1	25.4
Chest X-ray	NORMAL	Training	99.3	99.3	99.6
		ImageNet	97.6	100	95.7
		Open Images	97.0	99.8	94.3
		Random	55.7	54.4	54.8
	PNEUMONIA	Training	97.8	99.1	99.8
		ImageNet	60.0	75.3	72.3
		Open Images	62.8	79.8	68.0
		Random	45.0	46.1	44.1

**Table 2:** Fooling rates $R_{f}$ (%) of nontargeted UAPs against Inception V3 models trained from random initialization. ζ=8% for the skin lesions and chest X-ray image classifications, and ζ=16% for the OCT image classification. The column “UAP” indicates which input image set was used to generate the UAP, except for “Random”, which indicates random UAPs.

UAP/Medical Images	Skin Lesion	OCT	Chest X-ray
Training	92.7	74.5	45.9
ImageNet	50.0	75.3	22.2
Random	7.3	9.9	0.4

## Data Availability

The code and data used in this study are available from our GitHub repository: github.com/kztakemoto/Natural_UAP.

## Supplementary Materials

The following supporting information can be downloaded at: https://www.mdpi.com/article/10.3390/jimaging8020038/s1, File S1: Supporting Figures (Figure S1: Clean images and their adversarial examples generated using nontargeted UAPs from the training, ImageNet, and Open Images datasets, against Inception V3 model for the skin lesion image classifications; Figure S2: Clean images and their adversarial examples generated using nontargeted UAPs from the training, ImageNet, and Open Images datasets, against Inception V3 model for the OCT image classifications; Figure S3: Clean images and their adversarial examples generated using nontargeted UAPs from the training, ImageNet, and Open Images datasets, against Inception V3 model for the chest X-ray image classifications; Figure S4: Normalized confusion matrices for ResNet50 and VGG16 models attacked using nontargeted UAPs from ImageNet and Open Images datasets for skin lesions image classifications; Figure S5: Normalized confusion matrices for ResNet50 and VGG16 models attacked using nontargeted UAPs from ImageNet and Open Images datasets for OCT image classifications; Figure S6: Normalized confusion matrices for ResNet50 and VGG16 models attacked using nontargeted UAPs from ImageNet and Open Images datasets for chest X-ray image classifications) and Tables (Table S1: Composition of the predicted labels of ImageNet and Open Images datasets from Inception V3, ResNet50, and VGG16 models for skin lesion image classification; Table S2: Composition of the predicted labels of ImageNet and Open Images datasets from Inception V3, ResNet50, and VGG16 models for OCT image classification; Table S3: Composition of the predicted labels of ImageNet and Open Images datasets from Inception V3, ResNet50, and VGG16 models for chest X-ray image classification).
