# Supplementary material for: Natural Images Allow Universal Adversarial Attacks on Medical Image Classification Using Deep Neural Networks with Transfer Learning
Source: J Imaging. 2022 Feb 4;8(2):38. doi: 10.3390/jimaging8020038 (PMC8875959; doi:10.3390/jimaging8020038)
Supplement: Supplementary file 1 [file jimaging-08-00038-s001.zip › jimaging-1494140-supplementary.pdf]

Supplementary material of "Natural images allow universal adversarial attacks on medical image classification using deep neural networks with transfer learning"

Akinori Minagi[1], Hokuto Hirano[1], Kazuhiro Takemoto[1*]
1) Department of Bioscience and Bioinformatics, Kyushu Institute of Technology, Iizuka, Fukuoka 820-8502, Japan
**Corresponding author's e-mail: takemoto@bio.kyutech.ac.jp*

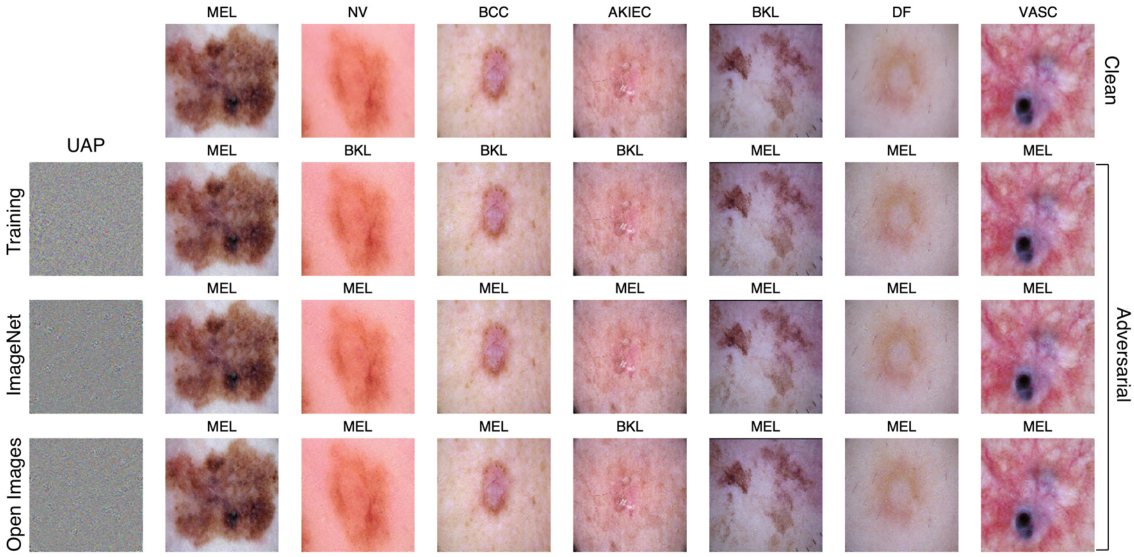

Figure S1: Clean images and their adversarial examples generated using nontargeted UAPs from the training, ImageNet, and Open Images datasets, against Inception V3 model for the skin lesion image classifications. Labels beside the images are the predicted classes. The clean (original) images are correctly classified into their actual labels.

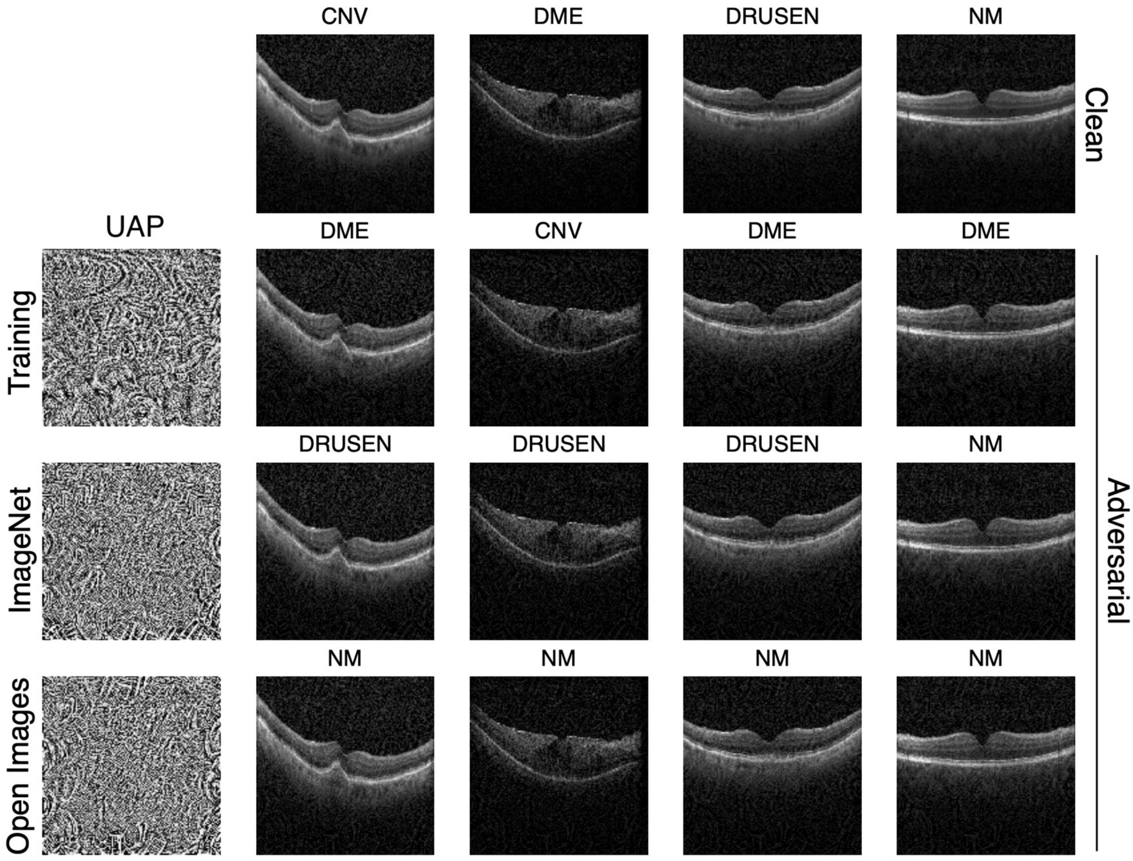

Figure S2: Clean images and their adversarial examples generated using nontargeted UAPs from the training, ImageNet, and Open Images datasets, against Inception V3 model for the OCT image classifications. Labels beside the images are the predicted classes. The clean (original) images are correctly classified into their actual labels.

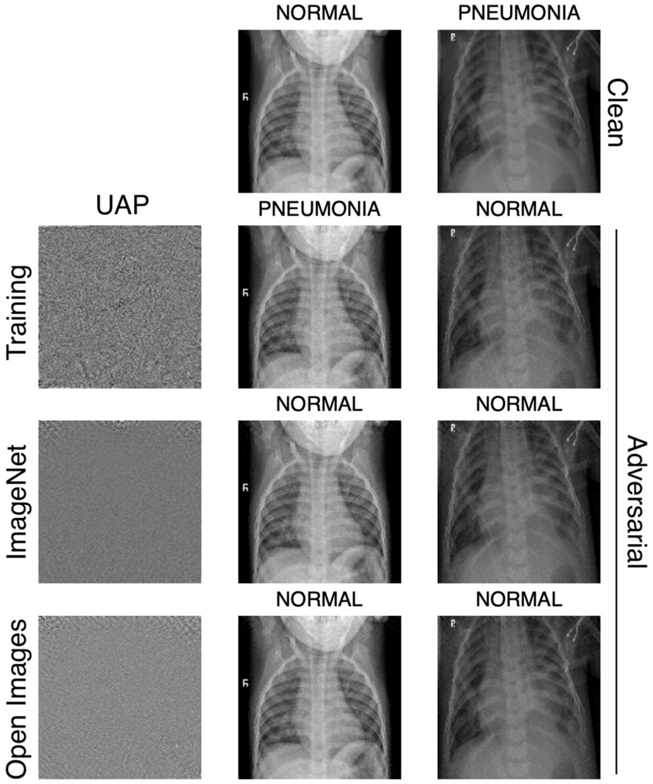

Figure S3: Clean images and their adversarial examples generated using nontargeted UAPs from the training, ImageNet, and Open Images datasets, against Inception V3 model for the chest X-ray image classifications. Labels beside the images are the predicted classes. The clean (original) images are correctly classified into their actual labels.

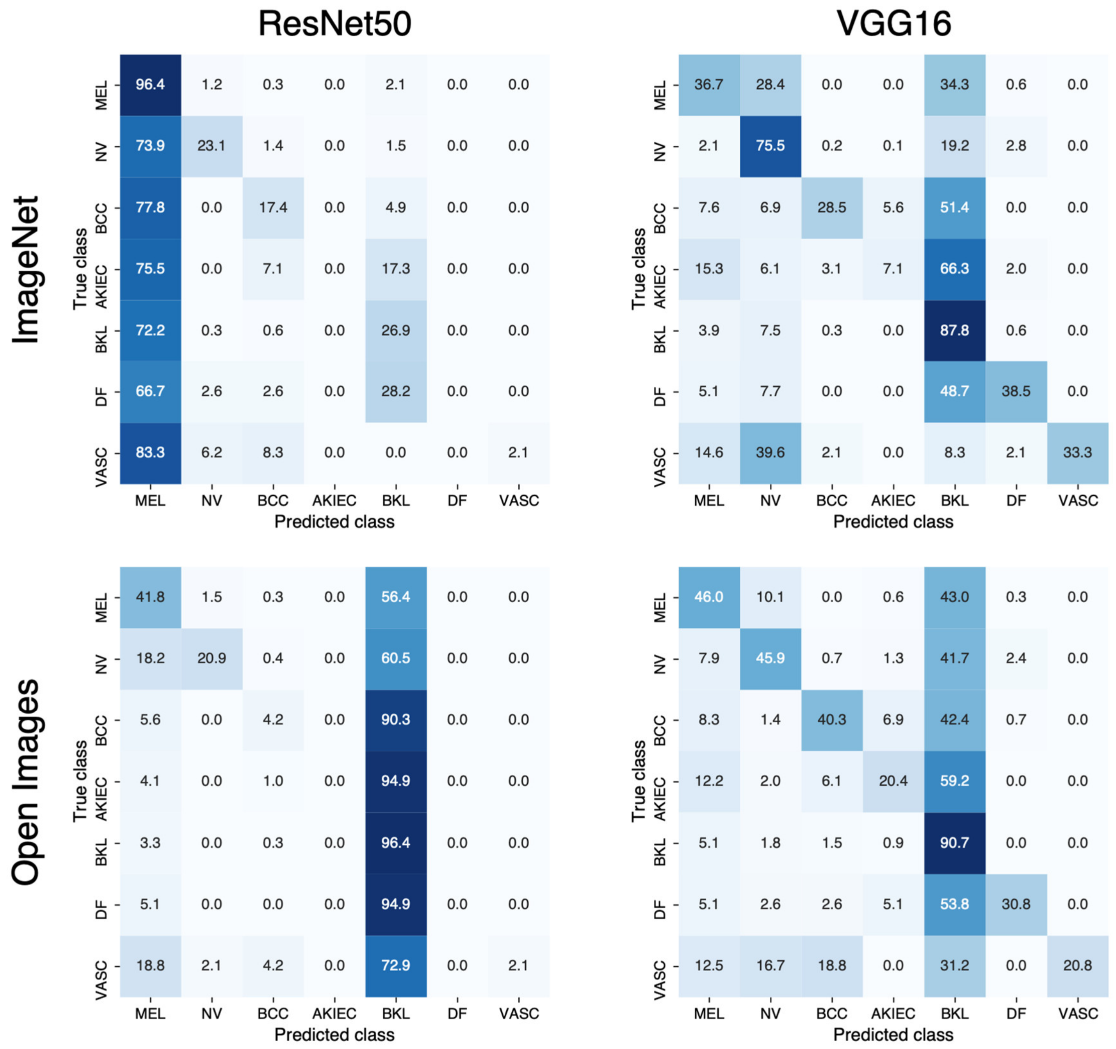

Figure S4: Normalized confusion matrices for ResNet50 and VGG16 models attacked using nontargeted UAPs from ImageNet and Open Images datasets for skin lesions image classifications.

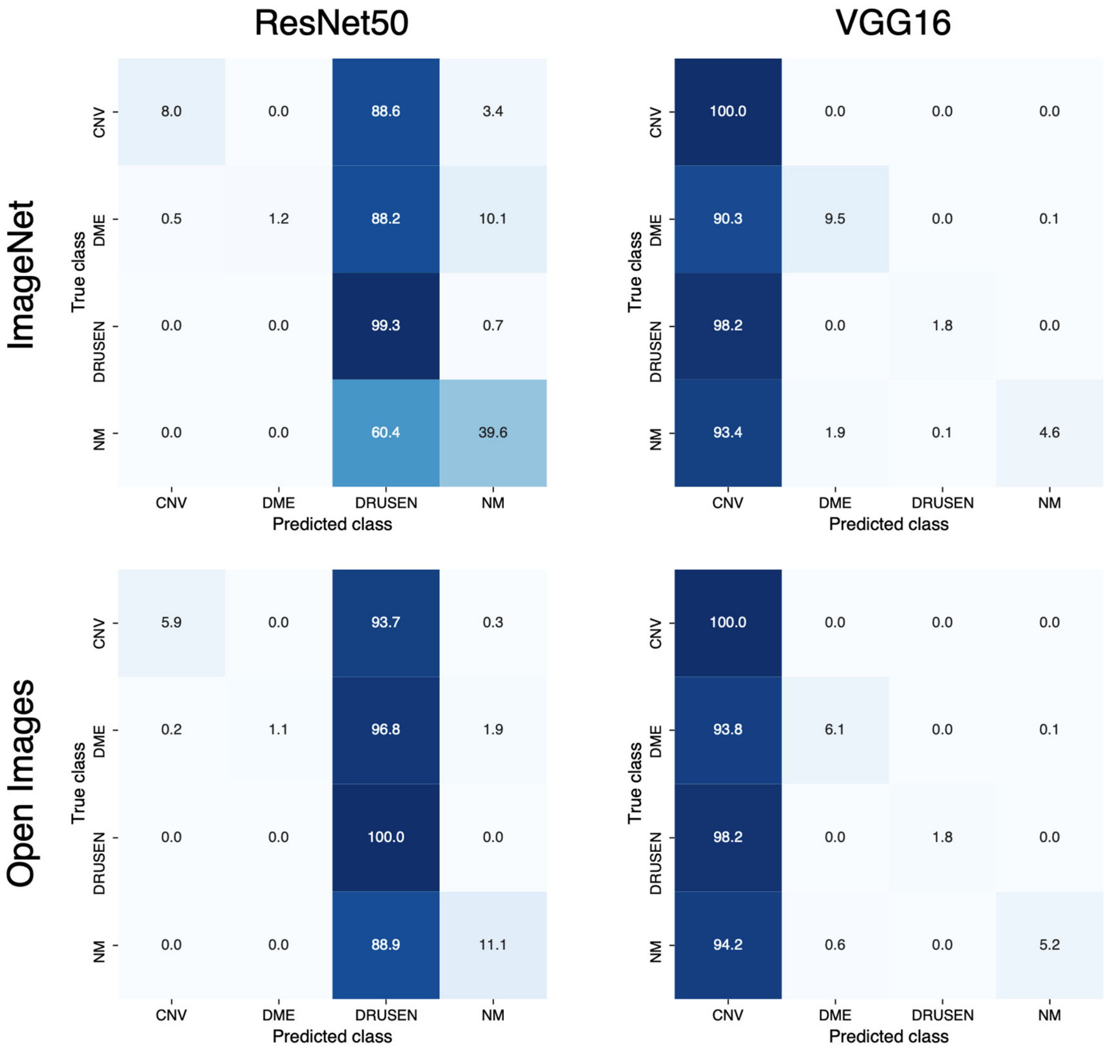

Figure S5: Normalized confusion matrices for ResNet50 and VGG16 models attacked using nontargeted UAPs from ImageNet and Open Images datasets for OCT image classifications.

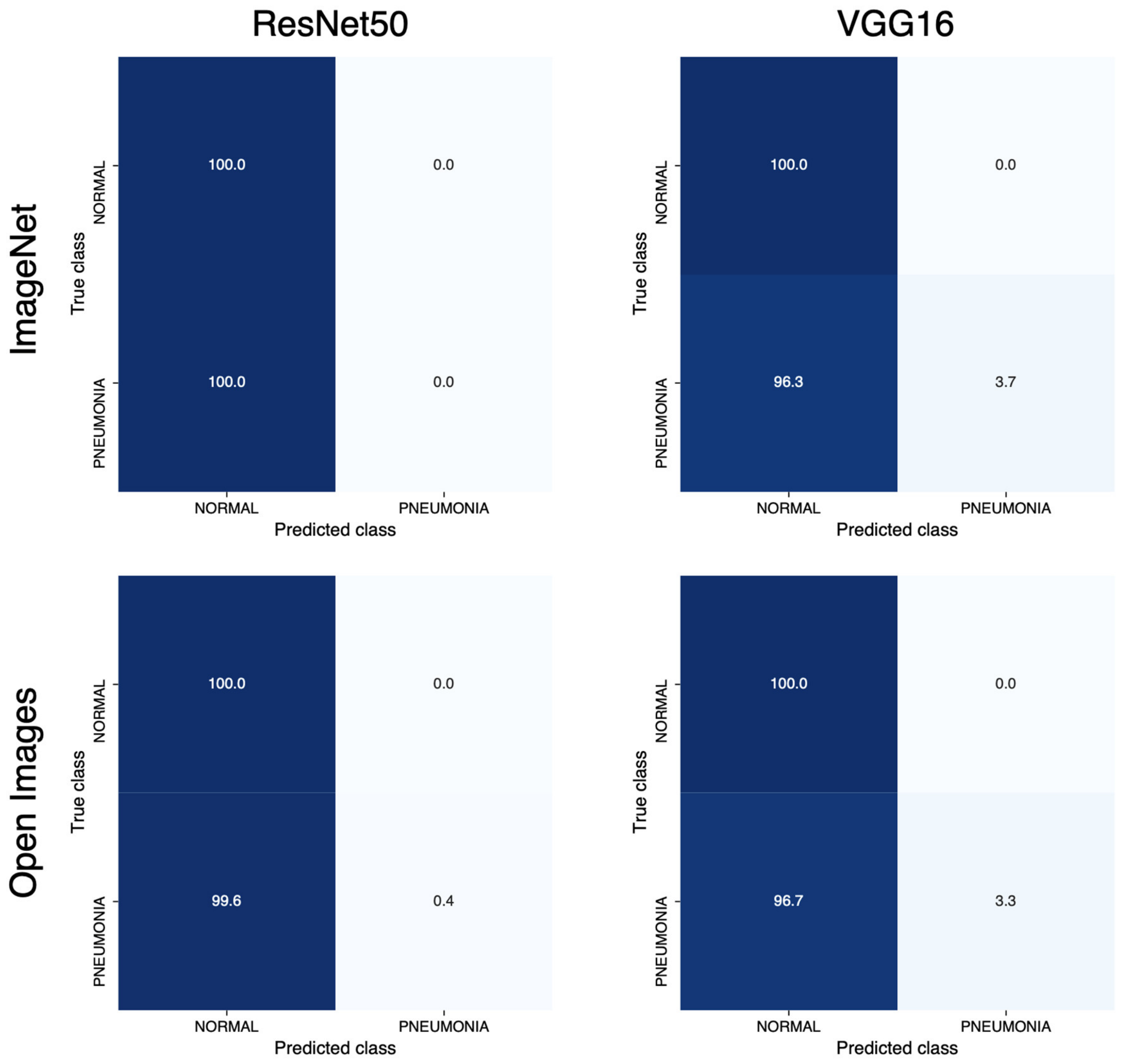

Figure S6: Normalized confusion matrices for ResNet50 and VGG16 models attacked using nontargeted UAPs from ImageNet and Open Images datasets for chest X-ray image classifications.

Table S1: Composition of the predicted labels of ImageNet and Open Images datasets from Inception V3, ResNet50, and VGG16 models for skin lesion image classification. Each dataset consists of randomly selected 100000 images.

Predicted labels	Inception V3		ResNet50		VGG16	
	ImageNet	Open Images	ImageNet	Open Images	ImageNet	Open Images
MEL	1722	803	14219	11776	5809	6768
NV	43649	41742	56716	66536	78906	76445
BCC	6556	7778	6471	4579	1844	2813
AKIEC	478	775	363	371	26	42
BKL	45563	46886	21709	15898	12730	12647
DF	459	285	109	112	151	226
VASC	1573	1731	413	728	534	1059

Table S2: Composition of the predicted labels of ImageNet and Open Images datasets from Inception V3, ResNet50, and VGG16 models for OCT image classification. Each dataset consists of randomly selected 100000 images.

Predicted labels	Inception V3		ResNet50		VGG16	
	ImageNet	Open Images	ImageNet	Open Images	ImageNet	Open Images
CNV	33705	27581	92188	90611	62837	64580
DME	53679	56899	1244	1267	34244	32294
DRUSEN	7075	9337	5201	6836	1685	1739
NM	5541	6183	1367	1286	1234	1387

Table S3: Composition of the predicted labels of ImageNet and Open Images datasets from Inception V3, ResNet50, and VGG16 models for chest X-ray image classification. Each dataset consists of randomly selected 100000 images.

Predicted labels	Inception V3		ResNet50		VGG16	
	ImageNet	Open Images	ImageNet	Open Images	ImageNet	Open Images
NORMAL	18157	14100	19910	24069	17208	18625
PNEUMONIA	81843	85900	80090	75931	82792	81375
